# Evaluating the Rhizosphere and Endophytic Microbiomes of a Bamboo Plant in Response to the Long-Term Application of Heavy Organic Amendment

**DOI:** 10.3390/plants11162129

**Published:** 2022-08-16

**Authors:** Xiaoping Zhang, Zhiyuan Huang, Zheke Zhong, Qiaoling Li, Fangyuan Bian, Guibin Gao, Chuanbao Yang, Xing Wen

**Affiliations:** 1 China National Bamboo Research Center, Key Laboratory of Bamboo Forest Ecology and Resource Utilization of National Forestry and Grassland Administration, Hangzhou 310012, China; 2 National Long-Term Observation and Research Station for Forest Ecosystem in Hangzhou-Jiaxing-Huzhou Plain, Hangzhou 310012, China; 3 Engineering Research Center of Biochar of Zhejiang Province, Hangzhou 310021, China

**Keywords:** bamboo forest, organic amendment, rhizosphere, plant endophyte, bacterial community

## Abstract

Root-associated bacteria play a major role in plant health and productivity. However, how organic amendment influences root-associated bacteria is uncertain in Lei bamboo (*Phyllostachys praecox*) plantations. Here, we compared the rhizosphere and endophytic microbiomes in two Lei bamboo plantations with (IMS) and without (TMS) the application of organic amendment for 16 years. The results showed IMS significantly increased (*p* < 0.05) the relative abundance of Proteobacteria and significantly decreased (*p* < 0.05) the relative abundance of Acidobacteria, Bacteroidetes, and Verrucomicrobiota. The root endophytic Proteobacteria and Acidobacteria were significantly higher in abundance (*p* < 0.05) in the IMS than in the TMS, while Actinobacteria and Firmicutes were significantly lower in abundance. Five taxa were assigned to Proteobacteria and Acidobacteria, which were identified as keystones in the rhizosphere soil microbiome, while two species taxonomically affiliated with Proteobacteria were identified as keystones in the root endophytic microbiota, indicating this phylum can be an indicator for a root-associated microbiome in response to IMS. The soil pH, soil total organic carbon (TOC), total nitrogen (TN), total phosphorus (TP), available potassium (AK), and TOC:TP ratio were significantly correlated (*p* < 0.05) with the bacterial community composition of both rhizosphere soils and root endophytes. TMS increased the microbial network complexity of root endophytes but decreased the microbial network complexity of rhizosphere soil. Our results suggest IMS shapes the rhizosphere and endophytic bacterial community compositions and their interactions differently, which should be paid attention to when designing management practices for the sustainable development of forest ecosystems.

## 1. Introduction

Bamboo forests play an important role in sequestering atmospheric CO_2_ for long-term storage in biomass because of their wide distribution, rapid growth, and high yields [1]. Lei bamboo (*Phyllostachys precox*) is a favored and widely-distributed species in southern China due to its edible shoot and the high economic returns associated with bamboo plantations. To obtain higher shoot yields and improve the economic benefits, intensive management practices, including excessive fertilization and use of surface mulch of organic residues, have been widely applied in bamboo plantations [2,3]. However, long-term intensive management can lead to several environmental and ecological issues, such as soil acidification [4] and the decreased stability of organic carbon [3]. To date, there have been no effective measures to solve these problems associated with the long-term intensive management of bamboo plantations. An in-depth understanding of the mechanism of the problems would facilitate the sustainable development of bamboo plantations.

Plants are colonized by a wide range of microorganisms that play essential roles in plant health and productivity [5]. Plant roots are the primary sites for plants to obtain nutrients from soil and exude organic molecules into the soil, thereby promoting plant–soil interactions [6]. Root-associated microbiota play an important role in plant health, nutrient acquisition and uptake, biomass production, and stress tolerance [7,8,9,10]. Previous studies have found that the management system influences root-associated microbial community compositions [11,12]. Longley et al. [11] found management practices affect whole-plant microbiomes, and specific indicator species varied between different managements. Hartman et al. [12] indicated that land management types and tillage intensities significantly affect dominant or well-connected microbes (bacteria and fungi) in soil and roots. However, most studies have mainly focused on the root-associated microbiota of model plant species [13,14] and major crops [15,16].

Therefore, in the present study, we comparatively investigated the effects of the long-term application of heavy organic amendment on soil properties and rhizospheric and endophytic bacterial communities in Lei bamboo plantations. The specific objectives of the study were to: (1) investigate the effects of long-term application of heavy organic amendment on soil physiochemical characteristics and C:N:P stoichiometry; (2) test the change in rhizospheric and endophytic bacterial communities of Lei bamboo under long-term application of heavy organic amendment; and (3) examine the relationships between soil physiochemical characteristics and enzymatic activities and the changes in the root-associated microbiome.

## 2. Results

### 2.1. Soil Physiochemical Characteristics and C:N:P Stoichiometry

The physicochemical characteristics of the selected soil samples are shown in Table 1. Compared with the TMS group, TOC, TN, TP, AK, C:P, and N:P were significantly (*p* < 0.05) increased in the IMS group, whereas pH was significantly (*p* < 0.05) decreased. However, no significant difference (*p* > 0.05) was observed between the two groups in C:N.

### 2.2. α-Diversity of Bacterial Communities

**Figure 1:**
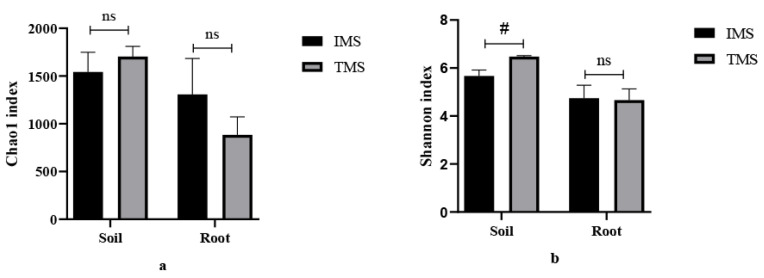
Difference in alpha diversity as measured by (**a**) Chao1 and (**b**) Shannon indices. IMS, intensive management system; TMS, traditional management system; #, *p* < 0.05; ns, not significant *p* > 0.05.

The alpha diversity indices (Chao1 and Shannon indices) of the bacterial communities are shown in Figure 1. For the rhizosphere soil bacterial communities, IMS showed a lower (*p* < 0.05) Shannon index compared with the TMS, but no differences between the two (*p* > 0.05) were observed for the Chao1 index. Among the root endophytic bacterial community structures, no significant (*p* > 0.05) change in the alpha indices (Shannon and Chao1) were found between the TMS and IMS groups.

### 2.3. Compositions of Bacterial Communities

**Figure 2:**
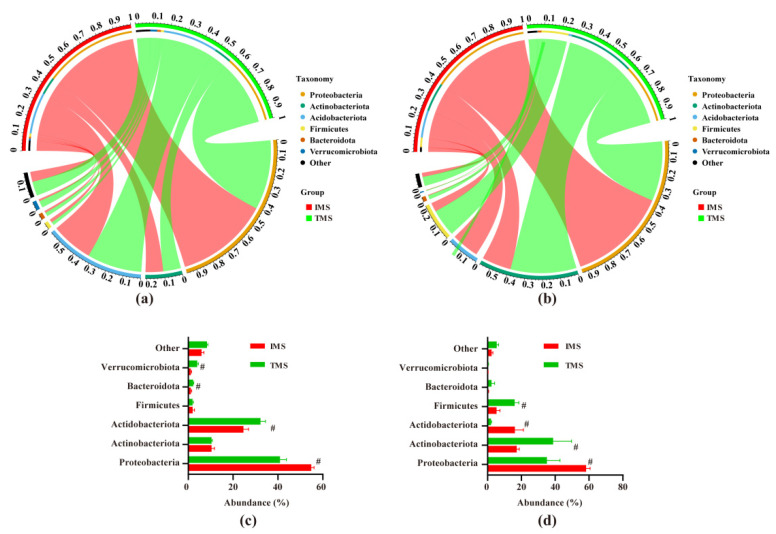
The distribution of the relative abundances of the dominant microbial phyla between the intensive management system (IMS) and traditional management system (TMS). (**a**,**c**) soil; (**b**,**d**) root. #, *p* < 0.05.

As shown in Figure 2, a total of six phyla had relative abundances of more than 1% across all samples, including Proteobacteria (47.28%), Actinobacteria (19.20%), Acidobacteria (18.74%), Firmicutes (6.30%), Bacteroidetes (1.53%), and Verrucomicrobiota (1.43%). Moreover, 16 bacterial genera with an average relative abundance >1% were found in the rhizosphere soil and root samples (Table S1), including *Streptomyces* (5.09%), Mycobacterium (4.83%), Chujaibacter (4.36%), *Burkholderia-Caballeronia-Paraburkholderia* (4.35%), *Acidibacter* (3.67%), *Acidothermus* (3.32%), *Acidipila* (2.69%), *Subgroup_13* (2.15%), *Occallatibacter* (2.07%), *Subgroup_2* (1.94%), *Acidocella* (1.64%), *Acidisoma* (1.60%), Bradyrhizobium (1.45%), Bacillus (1.42%), *Granulicella* (1.29%), and *Actinospica* (1.04%).

Individual taxa at the phylum and genus levels were compared using independent sample t-tests (Figure 2 and Table S1). For the rhizosphere soil bacterial communities, the relative abundance of Acidobacteria, Bacteroidetes, Verrucomicrobiota, *Bradyrhizobium*, *Occallatibacter*, *Subgroup_2*, *Streptomyces*, *Mycobacterium*, *Actinospica*, *Bacillus*, and *Burkholderia-Caballeronia-Paraburkholderia* were significantly decreased (*p* < 0.05), and that of Proteobacteria, *Acidipila*, *Subgroup_13*, *Acidothermus*, *Chujaibacter*, *Acidibacter*, *Acidocella*, and *Acidisoma* were significantly increased (*p* < 0.05) in the TMS samples compared with those of the IMS. In the root endophytic bacterial communities, Proteobacteria, Acidobacteria, Acidipila, Subgroup_13, Acidothermus, Actinospica, Chujaibacter, Acidibacter, Acidocella, and Acidisoma were significantly higher in the IMS samples, while Actinobacteria, Firmicutes, *Granulicella*, *Mycobacterium*, *Bacillus*, *Burkholderia-Caballeronia-Paraburkholderia*, and *Bradyrhizobium* were significantly lower than the TMS samples.

### 2.4. Factors Driving the Bacterial Communities

**Figure 3:**
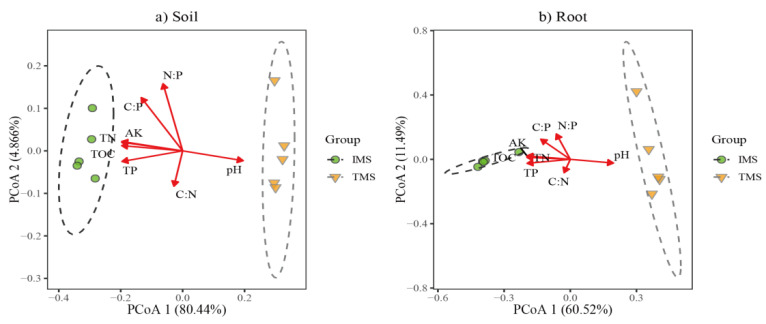
Principal coordinates analysis (PCoA) of bacterial communities in bamboo rhizosphere soil (**a**) and root (**b**). Vectors show fitted values of soil environmental parameters.

**Figure 4:**
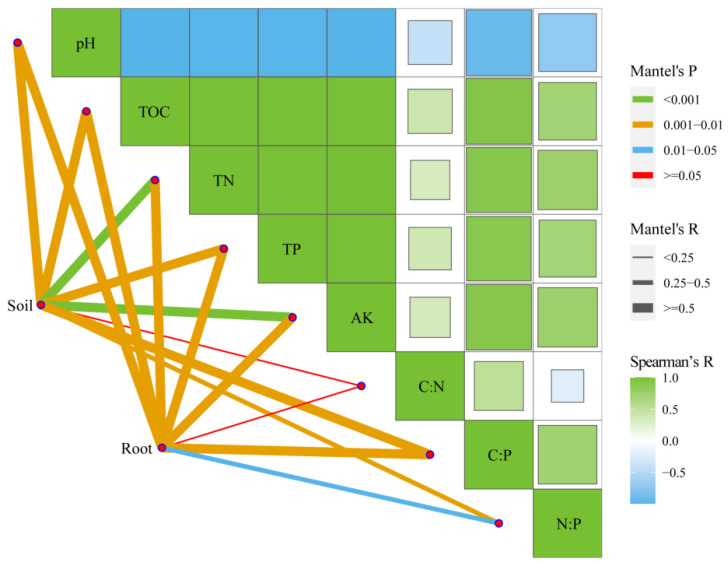
Spearman’s correlation analysis and Mantel tests for root-associated bacterial communities.

For rhizosphere soil bacterial communities, the first and second PCoA axes explained 80.44% and 4.86% of the community variation, respectively (Figure 3a). In addition to C:P (r^2^ = 0.855; *p* = 0.002) and N:P (r^2^ = 0.738; *p* = 0.011), five soil factors were significantly correlated with the PCoA ordination: pH (r^2^ = 0.988; *p* = 0.006), TOC (r^2^ = 0.990; *p* = 0.005), TN (r^2^ = 0.996; *p* = 0.002), TP (r^2^ = 0.992; *p* = 0.002), and AK (r^2^ = 0.997; *p* = 0.001). For root endophytic bacterial communities, the first two PCoA axes explained 60.52% and 11.49% of the community variation, respectively (Figure 3b). The fit analysis revealed that differences in the microbial structure were strongly correlated with soil pH (r^2^ = 0.965; *p* = 0.007), TOC (r^2^ = 0.969; *p* = 0.007), TN (r^2^ = 0.981; *p* = 0.002), TP (r^2^ = 0.987; *p* = 0.001), AK (r^2^ = 0.978; *p* = 0.004), and C:P (r^2^ = 0.741; *p* = 0.013). The Mantel test showed that soil pH, TOC, TN, TP, AK, C:P, and N:P significantly (*p* < 0.05) influenced rhizosphere soil and root endophytic bacterial community composition (Figure 4).

### 2.5. Properties of Microbial Co-Occurrence Networks

**Figure 5:**
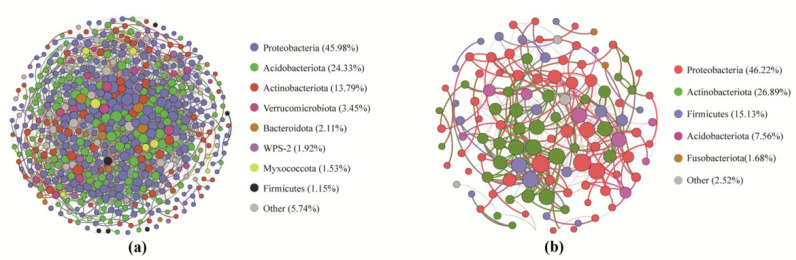
Co-occurrence networks of bacterial communities at OTU level colored by phylum. (**a**) soil; (**b**) root.

To evaluate the effects of IMS on bacteria–bacteria interactions in bamboo soils and roots, we structured a soil bacterial network based on correlations between ASVs. The rhizosphere soil and root endophytic bacterial community networks consisted of 522 and 119 nodes, along with 3105 and 330 edges, respectively (Figure 5). Their average path lengths (APL) were 3.687 and 3.767 with network diameters (ND) of 11 and 12, respectively, whereas the average degrees (AD) were 11.897 and 5.456, respectively. The modularity (MD) was 4.155 and 1.352, respectively. The average clustering coefficients (CC) were 0.365 and 0.397, respectively. The nodes in the soil network were mainly assigned to three bacterial phyla (Proteobacteria, Acidobacteria, and Actinobacteria), which accounted for 84.1% of all nodes. Five ASVs were identified as keystone taxa and taxonomically affiliated with Proteobacteria and Acidobacteria. For the root endophytic bacterial network, the nodes were assigned to five phyla (more than 1%): Proteobacteria, Actinobacteria, Firmicutes, Acidobacteria, and Fusobacteriota, which accounted for 97.48% of all nodes. Two ASVs were identified as keystone species and assigned to the phylum Proteobacteria.

To further investigate the microbial co-occurrence patterns within each soil and root sample, four networks were constructed based on the OTU level (Figure S1), and the network properties are summarized in Table 2. A co-occurrence network analysis showed that IMS decreased the nodes and edges of soil bacterial networks, indicating that long-term intensive management reduced the soil bacterial taxa numbers and their inner connections. The modularity values of the co-occurrence networks in all groups were higher than 0.4, suggesting that these bacterial networks had a modular structure [17]. Additionally, the negative correlation of the soil microbial networks in the IMS group was higher than that in the TMS group. Unlike the soil bacterial communities, IMS increased the amount of root microbiota, as evidenced by more nodes and edges of root microbial networks, and increased the positive interaction among the root microbes, as evidenced by the higher values of average degree, graph density, and negative correlation of root microbial networks than the TMS group.

## 3. Discussion

### 3.1. Influences of IMS on Soil Physicochemical Properties

In the current study, we found that IMS significantly increased rhizosphere soil TOC, TN, TP, and AK, and reduced soil pH. It can be expected that the high amount of long-term input of organic amendment (rice husk) increased the TOC and other soil nutrient elements [18]. However, the soil C:N ratio remained constant in this study. The total C and N contents of rice husks were 48% and 0.78%, respectively, and the C:N ratio was 61.5:1 [19]. Input C decomposed rapidly. A coupling relationship may exist between soil C and N, which shows a synchronous response to environmental changes, and the C:N ratio is mediated by soil microbes in a relatively stable state [20]. It is interesting to see that the pH decreased with the heavy organic amendment, which might be due to the accumulation of organic matter [21]. In addition, the decomposition of organic materials in soil can release CO_2_ [22], which has an acidifying effect (CO_2_ + H_2_O→H^+^ + HCO^3−^). IMS causes the soil C and N to increase rapidly in bamboo plantations [18], while the increase in P is relatively slow because of its different sources and relative stability [20,23]. These may contribute to the decreased soil C:P and N:P in the bamboo plantations after IMS. As above, long-term application of heavy organic amendment also leads to soil acidification and nutrient imbalance in the rhizosphere of Lei bamboo. 

### 3.2. Influences of IMS on the Bamboo Rhizosphere Soil Bacterial Communities Compositions 

A study indicated that intensive management (>15 years) strongly decreased bacterial α-diversity indices (phylogenetic diversity and OTU richness) in Moso bamboo forests [24]. Our study found that IMS significantly decreased the rhizosphere Shannon index but did not affect the Chao1 index. These may be related to the soil acidification [25] and high available nutrient (N, P and K) contents in IMS soils [26]. These changes indicate that prolonged application of heavy organic amendments had a negative effect on rhizosphere microbial community complexity and evenness, but not total species richness.

In this study, we found that IMS significantly increased the relative abundance of Proteobacteria and decreased the abundance of Acidobacteria. Members of Proteobacteria play important roles in the cycling of C, N, and other nutrients [27]. Fierer et al. [28,29] discovered that Proteobacteria taxa can grow fast under higher availability of C and N conditions. Acidobacteria play a major role in biogeochemical processes and the maintenance of ecological functions [30], and increased organic substrates and nutrients could reduce their abundance [31]. A network analysis was used to evaluate the microbial interactions. The results also revealed that Proteobacteria and Acidobacteria were dominant in the network, and keystone bacterial taxa belonged to these two phyla. Based on our results, shifts in the Proteobacteria and Acidobacteria were the main contributors to variations in soil C and N content. We also found that IMS significantly decreased the abundance of Bacteroidota and Verrucomicrobiota. Wang et al. [32] showed that the relative abundance of Bacteroidetes is associated with the soil’s total organic carbon, total nitrogen, and basal respiration. Several studies have shown that Verrucomicrobia can digest complex polysaccharides for growth [33] because they contain carbohydrate-active enzyme-related genes [34,35]. Verrucomicrobia species also participate in the nitrogen cycle, such as nitrogen fixation and partial denitrification [36,37]. Additionally, soil pH was significantly correlated with Acidobacteria, Proteobacteria, Bacteroidetes, and Verrucomicrobiota [38,39]. Overall, the shifts in microbes were an adaptive response to changes in soil pH and nutrients (especially C and N).

The co-occurrence network analysis indicated that IMS significantly reduced the complexity of the microbial co-occurrence network in the rhizosphere soil. Changes in soil factors have been reported to play important roles in determining microbial network complexity, such as pH and salinity [40,41]. Thus, the shifts in soil properties were related to changes in the microbial network complexity. Additionally, IMS had a more negative connection compared to the TMS group, indicating that competitive relationships increased within the bacterial communities [42].

### 3.3. Influences of IMS on the Root Endophytic Bacterial Communities Compositions 

We found the Chao1 and Shannon indices were not affected in the roots of Lei bamboo under IMS. Zhang et al. [43] found that the Chao1 index in the root endophytic bacterial community of Lei bamboo significantly increased with the increasing duration of heavy organic amendment application, but the Shannon index remained unchanged. Previous studies have indicated that soil physicochemical properties have an important effect on the alpha diversity of the endophytic microbiome [44,45,46]. Thus, these different results may be due to the different soil physicochemical properties in the two studied areas.

A sequence analysis showed that Proteobacteria predominated in the bamboo root samples, which is consistent with the findings reported by Zhang et al. [43,47]. An increase in the occurrence of Proteobacteria was also observed in the roots of the IMS group. Members of the phylum, Proteobacteria, contain a large number of taxa involved in plant symbiotic bacteria [48] and beneficial bacteria that can inhibit pathogenic bacteria [49]. Studies have reported that Proteobacteria taxa are associated with numerous metabolic strategies, such as nitrogen fixation and methylotrophy [50,51]. Moreover, both keystone species also belong to the phylum Proteobacteria. Thus, Proteobacteria plays a key role in the bamboo growth and metabolism of C and N. We also found that long-term intensive management increased Acidobacteria and decreased Actinobacteria and Firmicutes. Studies have indicated that Acidobacteria actively interact with plants and can act as plant growth-promoting bacteria [52,53]. Endophytic Actinobacteria contribute to plant nutrient uptake [54], prevent herbivores, and promote the biocontrol of pathogens to improve plant growth [55,56]. Firmicutes taxa have the potential to enhance plant stress tolerance, growth, and nutrient uptake [57,58]. Long-term intensive management also has some impact on bamboo, such as decreased internal nutrient cycling, damage to the growth and regeneration of bamboo, and increased risk of the occurrence and outbreak of leaf-eating insects [59]. Overall, the shifts in the root endophytes were a response to the change in soil nutrients and the pH caused by IMS. 

Furthermore, our results demonstrated that the bamboo root endophyte–endophyte correlation was altered after adoption of IMS, and the complexity of the bacterial community increased. Santolini and Barabasi [60] suggested that complex networks with greater connectivity are more robust to environmental disturbances than simple networks with lower connectivity. As shown in Table 1, IMS had higher soil nutrients, C:P, and N:P, as well as lower pH values. This result might imply that network structural complexity may be relevant to soil factors.

## 4. Materials and Methods

### 4.1. Sample Collection 

This study was carried out in Fuyang District (119°72 E, 30°05 N), Hangzhou, Zhejiang Province, China. This region has a subtropical monsoon climate with a mean annual sunshine duration of 1709.4 h. The mean annual temperature of the region is 16.2 °C, and the mean annual precipitation is 1452.0 mm. *P. praecox* had been planted in the cut-over land from a natural broad-leaved forest for nearly 20 years. The entire study area (approximately 10 ha) had similar initial site conditions. Two contrasting management systems were adopted for bamboo plantations, namely the intensive management system (IMS) and traditional management system (TMS). IMS involved application of organic amendment mulch to the soil surface in November or December to increase soil temperature and preserve soil moisture for the early sprouting of bamboo shoots and chemical fertilizer (as the normal treatment). Rice husk was used as an organic amendment. The annual input of organic amendment and chemical fertilizer (NPK15-15-15 fertilizer) were 40 kg/ha and 600 kg/ha, respectively [3]. The intensive management was implemented during the past 16 years. In contrast, no organic amendment was applied in TMS, but the other management measures were similar to those used for IMS. 

Five 10 × 10 m sampling plots along the diagonal of the plantation were established for the two plantations that adopted different management practices. In each sampling plot, bamboo roots from five bamboo plants were collected, and the roots were shaken to collect rhizosphere soils. Thereafter, the roots were washed with running tap water to remove adhering soil and surface sterilized according to Zhang et al. [43].

### 4.2. Analysis of Soil Physicochemical Properties and Enzyme Activities 

Soil pH was measured electrometrically using a soil-to-water ratio of 1:2.5. Soil total organic carbon (TOC) was analyzed using a TOC analyzer (Multi N/C 3100, Analytik Jena AG, Germany). Soil total nitrogen (TN, Kjeldahl method), total phosphorus (TP, using HClO_4_ and H_2_SO_4_ digestion), and available K (AK, extracted using 1 mol·L^−1^ ammonium acetate) were determined according to Lu [61]. 

### 4.3. DNA Extraction and Sequencing

The modified cetyltrimethylammonium bromide (CTAB) method was used to extract total community DNA from the soil and root samples. The concentration and purity of all extracted DNA was determined using 1%-agarose gel electrophoresis. Thereafter, the DNA was diluted with sterile water to a concentration of 1 ng μL^−1^. To minimize chloroplast contamination [62], the V5–V7 hypervariable regions of the 16S rRNA gene were amplified using the primers 799F (AACMGGATTAGATACCCKG) and 1193R (ACGTCATCCCCACCTTCC) [63,64] with sample-specific barcodes. The PCR reaction mixture contained 15 μL of 2 × Phusion Master Mix (Phusion^®^ High-Fidelity PCR Master Mix with GC Buffer; New England Biolabs, United States), 1 μL of template DNA, 2 μL of each primer (2 µM), and 10 μL of double-distilled H_2_O. The amplicons were generated using the following program: 98 °C for 1 min; 30 cycles of 98 °C for 10 s, 50 °C for 30 s, and 72 °C for 30 s; and 72 °C for 5 min. The PCR products were identified by 2%-agarose gel electrophoresis, and then purified using the GeneJET PCR Purification Kit (Thermo Fisher Scientific, Waltham, MA, USA). Library construction and high-throughput sequencing were performed by Novogene (Beijing, China) on the Illumina NovaSeq 6000 platform with PE250 per standard protocols.

### 4.4. Analysis of Sequencing Data

The data obtained on the Illumina NovaSeq 6000 platform were assigned to each sample based on their unique barcode sequences. After removing the barcode and primer sequences, the paired-end reads were merged using Fast Length Adjustment of Short reads (FLASH) [65] to obtain raw tags. Subsequently, fastp software [66] was used to perform quality control and obtain high-quality clean tags. All chimeric tags were removed, and effective tags were obtained for further analysis. The effective tags were imported into QIIME2 [67] and denoised with DADA2 [68] via the q2-dada2 plugin. Taxonomic assignments of the amplicon sequence variants (ASVs) were conducted using the qiime2-feature-classifier [69].

### 4.5. Statistical Analysis

Alpha indices (Chao1 and Shannon indices) were calculated using the ‘microeco’ R package [70]. Principal coordinates analysis (PCoA) with the envfit function method was conducted using the ‘vegan’ R package [71]. Mantel tests were used to evaluate the linkages between soil factors and bacterial community compositions, and the results were combined using the ‘vegan’ [71] and ‘ggcor’ [72] packages in R. OTUs in >80% of each treatment were selected to construct the co-occurrence network. Spearman’s correlation was estimated using the ‘WGCNA’ package [73] in R. The microbial co-occurrence networks were built based on robust correlations (Spearman’s correlation coefficient > 0.6 and FDR-adjusted *p* < 0.05) using the ‘igraph’ package in R [74] and visualized using Gephi software [75]. The keystone OTUs were defined as nodes within the top 1% of node degree values of each network; these OTUs were identified separately for rhizosphere soil and root meta-networks.

## 5. Conclusions

This study demonstrated that a decrease in pH and increase in TOC and other nutrients were observed in the rhizosphere soil of Lei bamboo under IMS, indicating that IMS also leads to acidification and nutrient imbalance in rhizosphere soil. IMS can affect the rhizosphere soil properties as well as the root-associated bacterial community structures, such as increasing the microbial network complexity of rhizosphere soils but decreasing the microbial network complexity of endophytes. Proteobacteria can be an indicator for root-associated microbiomes in bamboo forest exposed to IMS. Moreover, changes in both rhizospheric and endophytic bacterial communities were significantly correlated with pH, TOC, TN, TP, AK, and C:P. Our findings provide a better understanding of the effects of IMS on the rhizosphere soil properties and root-associated bacterial communities in bamboo plantations and could be used in designing improved bamboo ecosystems.

## Figures and Tables

**Table 1:** Soil properties in bamboo rhizosphere soils from an intensive management system (IMS) and traditional management system (TMS).

	IMS	TMS
pH	4.47 ± 0.01 b	4.74 ± 0.01 a
TOC (g/kg)	87.54 ± 2.96 a	36.92 ± 0.92 b
TN (g/kg)	6.22 ± 0.17 a	2.73 ± 0.13 b
TP (g/kg)	0.95 ± 0.03 a	0.46 ± 0.02 b
AK (mg/kg)	296.82 ± 6.06 a	79.16 ± 5.21 b
C:N	14.09 ± 0.51 a	13.55 ± 0.92 a
C:P	92.01 ± 3.24 a	80.42 ± 2.67 b
N:P	6.53 ± 0.14 a	5.96 ± 0.41 b

Different lowercase letters within rows indicate significant difference (*p* < 0.05).

**Table 2:** The topological features of bacterial networks associated with rhizosphere soils and roots of Lei bamboo.

	Soil		Root	
	IMS	TMS	IMS	TMS
Nodes	1064	1232	381	222
Edges	6419	8225	1643	462
Average degree	12.066	13.352	8.625	4.162
Modularity	1.389	1.375	1.354	3.631
Graph density	0.011	0.011	0.023	0.019

## Data Availability

The sequencing data have been deposited in NCBI under Bioproject PRJNA867534 and PRJNA867541.

## Supplementary Materials

The following supporting information can be downloaded at: https://www.mdpi.com/article/10.3390/plants11162129/s1, Figure S1: Bacterial networks in bamboo rhizosphere soil (a, b) and root (c, d) collected from intensive management system (IMS) and traditional management system (TMS); Table S1: Comparative analysis for the relative abundance of dominant bacterial genera in rhizosphere soils and roots of Lei bamboo.
