# Supplementary material for: Evaluating the Rhizosphere and Endophytic Microbiomes of a Bamboo Plant in Response to the Long-Term Application of Heavy Organic Amendment
Source: Plants (Basel). 2022 Aug 16;11(16):2129. doi: 10.3390/plants11162129 (PMC9412275; doi:10.3390/plants11162129)
Supplement: Supplementary file 1 [file plants-11-02129-s001.zip › plants-1836822-supplementary.pdf]

Table S1. Comparative analysis for the relative abundance of dominant bacterial genera in rhizosphere soils and roots of Lei bamboo.

**Phylum**	**Genus**	**Soil**		**Root**	
		**IMS**	**TMS**	**IMS**	**TMS**
Acidobacteria	*Acidipila*	4.02% ± 0.52%a	1.29% ± 0.13%b	5.37% ± 3.01%a	0.08% ± 0.03%b
Acidobacteria	*Subgroup_13*	5.78% ± 0.68%a	2.48% ± 0.31%b	0.27% ± 0.12%a	0.06% ± 0.06%b
Acidobacteria	*Occallatibacter*	2.32% ± 0.31%b	5.66% ± 0.50%a	0.16% ± 0.05%a	0.16% ± 0.07%a
Acidobacteria	*Subgroup_2*	2.05% ± 0.22%b	5.45% ± 0.94%a	0.10% ± 0.07%a	0.14% ± 0.11%a
Acidobacteria	*Granulicella*	0.91% ± 0.19%a	0.83% ± 0.10%a	2.73% ± 1.47%a	0.69% ± 0.49%b
Actinobacteria	*Streptomyces*	0.11% ± 0.17%b	0.95% ± 0.53%a	1.19% ± 0.61%a	18.12% ± 13.86%a
Actinobacteria	*Mycobacterium*	0.54% ± 0.08%b	1.09% ± 0.38%a	2.51% ± 1.00%b	15.17% ± 4.37%a
Actinobacteria	*Acidothermus*	4.07% ± 0.34%a	2.68% ± 0.17%b	5.70% ± 1.98%a	0.82% ± 0.23%b
Actinobacteria	*Actinospica*	0.38% ± 0.09%b	0.55% ± 0.09%a	3.07% ± 1.43%a	0.16% ± 0.11%b
Firmicutes	*Bacillus*	0.33% ± 0.15%b	0.58% ± 0.17%a	0.86% ± 0.61%b	3.90% ± 1.21%a
Proteobacteria	*Chujaibacter*	13.47% ± 2.38%a	1.85% ± 0.15%b	2.03% ± 0.78%a	0.07% ± 0.02%b
Proteobacteria	*Burkhold-eria-Caballeronia-Paraburkholderia*	1.00% ± 0.21%b	2.29% ± 0.50%a	2.45% ± 1.47%b	11.66% ± 4.72%a
Proteobacteria	*Acidibacter*	6.89% ± 0.46%a	3.68% ± 0.32%b	2.89% ± 0.94%a	1.21% ± 0.68%b
Proteobacteria	*Acidocella*	0.15% ± 0.06%a	0.01% ± 0.01%b	6.31% ± 1.95%a	0.09% ± 0.04%b
Proteobacteria	*Acidisoma*	0.23% ± 0.08%a	0.11% ± 0.03%b	5.97% ± 0.97%a	0.10% ± 0.04%b
Proteobacteria	*Bradyrhizobium*	1.05% ± 0.11%b	2.42% ± 0.32%a	0.59% ± 0.19%b	1.72% ± 0.49%a
Proteobacteria	*KF-JG30-C25*	1.79% ± 0.09%a	1.95% ± 0.16%a	0.15% ± 0.09%a	0.10% ± 0.09%a

Different lowercase letters within rows indicate significant difference ($p < 0.05$). IMS, intensive management system; TMS, traditional management system.

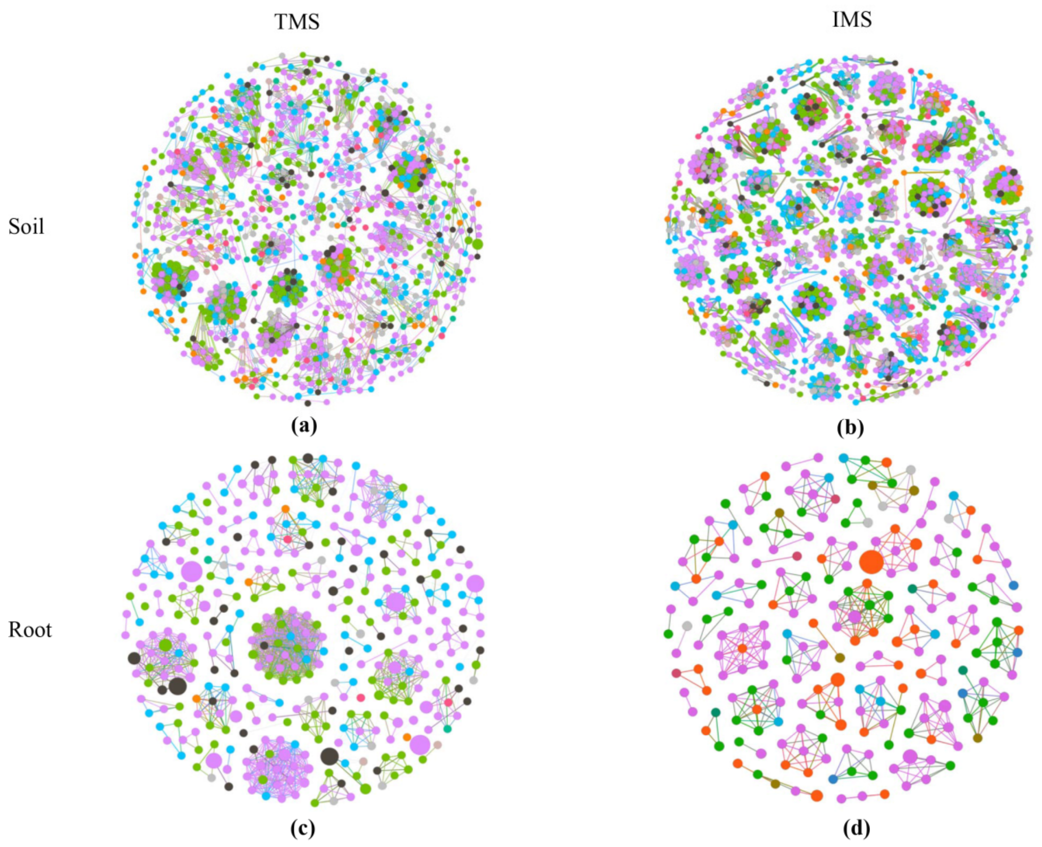

Figure S1. Bacterial networks in bamboo rhizosphere soil (a, b) and root (c, d) collected from intensive management system (IMS) and traditional management system (TMS).
